# Parameter estimations of sigmoidal models of cancer

**DOI:** 10.1186/1471-2105-15-S10-P15

**Published:** 2014-09-29

**Authors:** Ferhan M Atici, Mustafa Atici, Ngoc Nguyen

**Affiliations:** 1 Western Kentucky University, Department of Mathematics, Bowling Green, KY 42101-3576, USA; 2 Western Kentucky University, Department of Computer Science, Bowling Green, KY 42101-3576, USA

## Background

Tumor growth, a relationship between tumor size and time, is of special interest since growth estimation is very critical in a clinical practice. There are some mathematical models which describe tumor growth and have prediction capabilities. Typically, there are three ways to model non-complex growth behavior: exponential, logistic and sigmoidal. In 1825, Benjamin Gompertz introduced the Gompertz function, a sigmoid function, which is found to be applicable to various growth phenomena, in particular tumor growth. Besides the Gompertz model which includes three parameters, the Weibull and Richards models with four parameters are known as sigmoidal models.

The aim of this project is to introduce continuous fractional and discrete fractional models of the tumor growth and also estimate parameters of these models in order to have better data fitting.

## Materials and methods

We outline approximation techniques to choose the appropriate exponential functions of discrete and continuous fractional calculus [1]. We demonstrate how to replace the exponential function *e^-ct^* in the existing continuous time models with the exponential functions of fractional calculus. We compare continuous, discrete, continuous fractional and discrete fractional forms of these sigmoidal curves by using the tumor growth data for twenty-eight mice [2]. These controlled mice were inoculated with tumors but did not receive any succeeding treatment. We apply residual sum of squares and cross-validation methods to compare models on data fitting and predictive performances. Estrus cycle stages of measurement are also taken into account when comparing the models.
